# Video versus live lecture courses: a comparative evaluation of lecture types and results

**DOI:** 10.1080/10872981.2018.1555434

**Published:** 2018-12-17

**Authors:** Thomas Brockfeld, Bringfried Müller, Jan de Laffolie

**Affiliations:** a General Pediatrics & Neonatology, Justus-Liebig-University, Giessen, Germany; b Medi-Learn, Private Institute Offering Medical Revision Courses For Medical Students, Marburg, Germany

**Keywords:** Lecture videos, clinical education, educational technologies, video-based teaching

## Abstract

**Background**: Video lectures are an increasingly popular format. They allow an individual choice of time, place and speed of learning.

**Objective**: The aim of the present study was to compare whether video lectures are as effective as live lectures to impart the complete contents of the clinical part of the medical exam. The study also examines whether students prefer live or video lectures and for what reason.

**Design**: In 2014, a preparatory course was held at the University of Göttingen to train medical students for the clinical part of the medical exams. Three-quarters of the participants received 41 four-hour lessons live, while the same lessons were shown on video to the remaining quarter. The assignment to the video group changed daily, so that all students saw both live and video lectures. To compare the effectiveness, it was evaluated for 205 students how video and live students answered the 301 multiple choice questions of the medical exam.

**Results**: There is hardly any difference regarding effectiveness. 36,735 of 46,926 questions (78.283%) were correctly answered by the *live group,* while 11,617 of 14,779 questions (78.605%) were correctly answered by the *video group* (n.s., *p* = 0.407, effect size ω = 0.003337). There were some differences in subjective evaluation: 48% of students preferred live lessons, 27% preferred video lessons and 25% stated ‘neutral’. The items ‘learning atmosphere’, ‘ability to concentrate’, ‘presence of other students’ and ‘acoustic intelligibility’ were assessed significantly better for the video courses than for the live courses. No item of the live course was rated better than in the video course.

**Conclusions**: Video and live lectures are equally effective in preparation for the clinical part of the medical exams. Video lectures offer many benefits for the students and for the faculties, and may complement and partly replace conventional live events.

## Introduction

Video recordings of lectures offer various benefits to the user. He can repeat the lecture subsequently at any time and place [1]. Students might gain time because the way to the lecture hall is no longer necessary [2].

**Figure 3.**
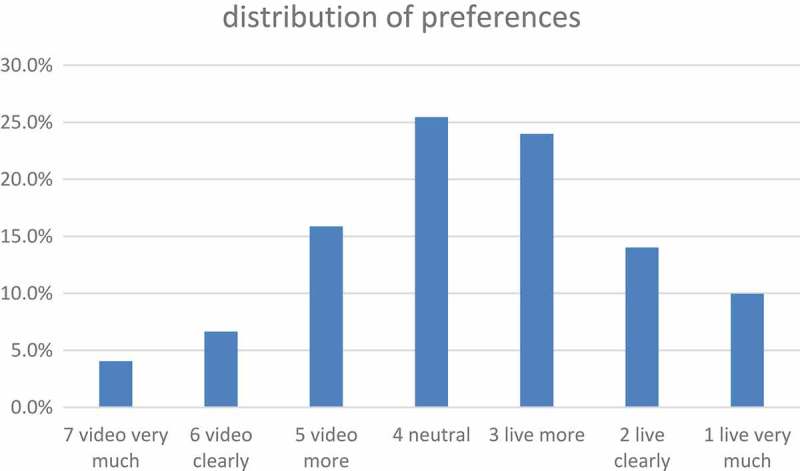
Distribution of preferences.

The speed in which the lecture proceeds can be determined by the student himself [3]. This is also true for self-paced learning [4]. Video lectures can be repeated as often as wanted, which is particularly useful for a deep understanding or for the preparation of exams [5].

Paegle et al. [6] compared the effect of live and video lectures on pathology. They found no significant differences in test questions (*n* = 59 4th-year medical students, 129 multiple-choice questions, average score and standard deviation live/video 87.56 (+4.80) vs 87.99 (+6.46)). Subjectively, however, the students thought they had learned more from the live lectures.

Schreiber et al. [7] came to a similar conclusion: In a test after 15-min sequences on the subjects of ‘vasculitis’ and ‘arthritis’, the video and the live group were equally good (*n* = 66 medical students, 34 multiple-choice questions, live/video 90.2% vs 87.8%, *p* = 0.15). But while 88% of the students rated the live performance as very good, the video presentation was rated equally well by only 62%.

Spickard et al. [2] also found out that, objectively, the test results for the students after a 1-h lecture were equally good.

Ramlogan et al. [8] came to a different conclusion. They offered three almost 15-min sequences each live and on video. The participants of the live lesson had significantly better results in a test than the participants of the video lesson (*n* = 85 students of dentistry, average score and standard deviation live/video 74.9 (+14.9) vs 68.6 (+16.3)). In their subjective assessment, however, 97% of the participants felt an improvement of their clinical abilities by the videos. Only 78.8% felt an improvement of their clinical abilities by the live lessons.

In a study about the use of video lectures in pharmacology, Fernandes et al. [9] found better test results for the visitors of the live event than for participants of the video lecture.

Most of the studies that compared live and video lectures in the medical field focused on a quite restricted part of the medical curriculum and use only a small number of test questions for the comparison of live and video lessons.

For example, Paegle et al. [6] showed a total of six lectures of 45–50 min each on video, Spickard et al. [2] presented a 1-h lecture and Schreiber et al. [7], as well as Ramlogan et al. [8], had two or three almost 15-min video sequences. Accordingly, only a few number of test questions were used for the comparison: Paegle et al. [6] used 15–25 test questions for each of their lectures, Spickard et al. [2] used four discussion questions and Schreiber et al. [7] put 15–19 questions for both of their lectures.

The purpose of this study is to evaluate whether video lectures for exam candidates for the second section of the medical examination have the same effect on the test results as live lectures. In addition, it is supposed to be investigated how the exam candidates evaluate the video compared to the live lectures.

What is new is that not only the effect of video and live courses as teaching methods is studied on a few, selected subjects, but that a comparison is made about the *complete* subject matter of the clinical section of the medical examination.

The following questions are supposed to be answered by the study:

- Do students gain the same results in their exams after taking part in video lectures as they do after attending live lectures?
- Are video lectures and live lectures subjectively assessed as equal by the students?
- How are video lectures perceived compared to live lectures regarding the learning atmosphere, the ability to concentrate, the usefulness for examinations, the intelligibility, the clarity and the tempo? How fascinating are video compared to live lectures?

## Methods

In Germany, the examination for the second section of the medical state examination is a uniform multiple-choice test (Type A questions), which is held twice a year at all medical faculties. A total of 320 exam items enclose the clinical fields as well as pharmacology, pathology, forensic medicine, social medicine and occupational medicine. The examination is passed if at least 60% of the questions have been answered correctly.

In the spring of 2014, MEDI-LEARN was entrusted by the University of Göttingen to conduct a 41-day course to prepare students for the second part of the medical examination. Each day includes 4 h of lecture.

A total of 296 students were registered for the Göttingen MEDI-LEARN course. As the largest available lecture hall was only designed for 272 listeners, the lecture had to be shown in parallel in a second lecture theater.

For this purpose, MEDI-LEARN has recorded all teaching units on video before. The lecturers were asked to give the same lecture they usually give in the live course. In almost every case, the lecturers of the live course could be won for the video recording, exceptions were the two cardiology and the infectiology videos. In the background, the same PowerPoint® presentation was displayed that later was shown on a monitor (SmartBoard®) in the live course. The lecturers were shown from the head to the hip in the picture; next to the lecturer, the monitor with the presentation was visible.

When the course scripts were dispersed at the start of the course, the students were distributed randomly to four counters. The groups should later be assigned either to live or video lectures.

At the introductory event, participants were given their timetable. Each lesson was offered simultaneously in two lecture halls: once live and once on video. Thus, the video could be shown under nearly equal conditions as the live lessons. The following features were the same for both events:

- the lecturer (exceptions see above)
- date, time and approximate duration of the lecture
- the Powerpoint® presentation used
- accompanying lecture notes
- transmission of the sound via the audio system of the lecture hall
- breaks

Since most lecturers held two or more classes, the students often saw a topic of the lecturer live, another topic of the same lecturer on video. On each course day, three of the four groups saw the live lecture, while one group attended the video lecture. On the first day of the course, group 1 saw the video, on the second day group 2 and so on.

**Figure 1.**
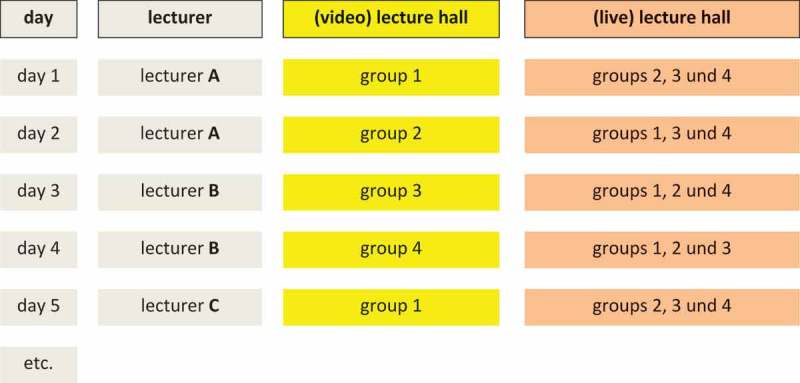
Crossover setting.

As a result, each group saw a quarter of the lectures on video and three quarters live, with each group having seen a different course on video. In turn, this crossover setting was also used to evaluate every day of the lessons, both by video and live participants (Figure 1. Crossover setting).

The Second Section of the Medical Examination takes place nationwide. The questions are available to the examining candidates in printed form. The solutions must be marked on a special computer sheet. The computer sheet must be returned until the processing time has expired. Students can take the task book with them after the exam.

The official results will be published only 2–3 weeks after the test. MEDI-LEARN offers a statistical evaluation in order to provide a first solution overview. To this end, a tool is offered to all students via internet, through which they can enter their solutions. As a result, the user can see for every exam question which solution the majority of students have chosen. On this basis, it is already possible to anticipate the test result.

Göttingen students were asked whether they had taken part in the course and which group from 1 to 4 they had been distributed to. Then, we have assigned the exam questions to each lecture day. Thus, we were able to determine for each student what question had been dealt with in a video or in a live lecture.

Since there were three groups in the live class and one group in the video class every day, each question was answered by three quarters of the students after a live lecture and of one quarter after a video lecture.

In order to avoid that a comparison of the results of the live and the video group was influenced by the composition of the video group, the video group changed on each day of the class. This ensured that all students answered questions on both video and live lessons.

**Figure 2.**
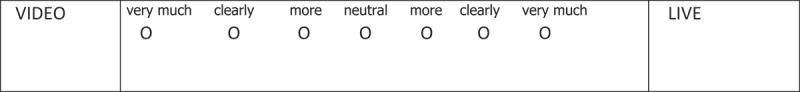
Bipolar rating scale.

The preference for the video or live course can be specified on a bipolar rating scale (Figure 2).

In addition, both live and video course should be assessed differently on a 6-point scale (1 = ‘very good’, 6 = ‘very poor’) according to the following criteria:

- learning atmosphere
- ability to concentrate
- presence of other students
- usefulness for the written examination
- usefulness for the oral examination
- structure
- intelligibility of content
- clarity of presentation
- degree of interest aroused
- tempo
- optical discernibility
- acoustic intelligibility
- lecture script
- the lecture overall

The data were statistically evaluated with the software SPSS 24.

To test for independence of the characteristics correct/false and live/video readings, we performed a Chi-square test, *p* value ≤ 0.05 significantly.

In the study of the preference, we performed a Wilcoxon signed rank test on a sample, *p* value ≤ 0.05, to assess the significance.

Since many items (and therefore hypotheses) were tested, we performed a Bonferroni correction and an α of $\frac{\alpha }{number\;of\;tested\;hypotheses}=$ $\frac{0,05}{14}$ = 0.0036 was chosen as a prerequisite for rejection of the null hypothesis.

## Results

Three hundred and one out of 320 questions could be assigned to the teaching units presented in the course. Of the 205 study participants, all the answers to these 301 questions are available, and it is known which of these contents were presented as part of a live video interview and which were taught in a video lecture.

Thus, of the total of 61,705 questions, the contents of 14,779 questions were conveyed to the participants in a video lecture, while the contents of 46,926 questions were presented in a live lecture.

A total of 36,735 of 46,926 questions (78.283%) were correctly answered by the live group, while 11,617 of 14,799 questions (78.605%) were correctly answered by the video group.

The video students of this study were on average 0.332 percentage points better – the difference was therefore very small.

Table 1 shows the distribution of correct and incorrectly solved questions on live and video lectures.

**Table 1.** Distribution of correct solutions for live and video groups.

	Right	Wrong	
LiveLive	**36,735**	**10,191**	46,926
Video	**11,617**	**3162**	14,779
	48,352	13,353	61,705

**Table 2.** Percentage of preference distribution.

		Frequency	Percent	Cumulative percentages
Valid	7 video very much	11	4.1	4.1
	6 video clearly	18	6.6	10.7
	5 video more	43	15.9	26.6
	4 neutral	69	25.5	52.0
	3 live more	65	24.0	76.0
	2 live clearly	38	14.0	90.0
	1 live very much	27	10.0	100.0
	Total	271	100.0	

One hundred and thirty out of 271 (48%) prefer live lessons, 69 (25%) rated ‘neutral’ and 72 (27%) prefer the video lessons. This shows that the students obviously prefer live lessons (see Figure 3 and Table 2).

In the differentiated course evaluation, different picture emerges than in the case of preference: Here, the characteristics learning atmosphere, concentration capacity, presence of other students and the acoustical intelligibility in the video lectures are assessed significantly better than in the live lectures, but vice versa no feature of the live lectures is assessed better than in the video lectures.

## Discussion

Previous studies have produced contradictory results regarding the effect of video readings compared to live lectures.

Paegle et al. [6] found only a difference of 0.43 percentage points in a test with a total of 129 MC questions when comparing video and live lectures. Schreiber et al. [7] used 35 questions and found only the slight difference of 2.4 percentage points. In the test by Spickard et al. [2], a maximum of 16 points could be reached. Here, the difference between live and video group was only 0.1 points. Solomon et al. [10] and Davis et al. [11] also found no significant difference.

In contrast to this, Ramlogan et al. [8] found significantly better results in the live group than in the video group: the difference in the posttest after the course was 6.3 percentage points (*p* = 0.049 in the variance analysis). However, the results were reached after a lesson in dental examination techniques. Perhaps, the clinical practical topic of this study explains that the live listeners performed better.

Previous studies about the efficacy of medical video lectures were only on few, selected subjects. Paegle et al. [6] compared the effect of six approximately 45-min lectures about gynaecological pathology, Schreiber et al. [7] referred to two lectures on rheumatology, each 15 min long and Spickard et al. [2] referred to a short lecture on ‘Evidence-Based Medicine’.

The present study is based on more than 160 lectures on the almost complete contents of the clinical section of the medical examination. It was also confirmed with this range of material that live and video lectures have the same effect on the examination performance.

For an α = 0.05 and an assumed β of 0.2, an effect strength of only ω = 0.012128 could have been measured at *n* = 61,705. The effect strength in this study was ω = 0.003337 significantly smaller. If there is a difference between live and video students, it is negligible.

In the subjective evaluation of the students, the live course was judged better than the video course in most studies. In Paegle et al. [6], students assessed the live course on a 9-point scale at 7.37, while the video was rated at only 5.93 (*p* <0.0003). In Spickard et al. [2], 96% were satisfied with the live course, but only 81% with the video course (*p* = 0.03). Schreiber et al. [7], as well as Solomon et al. [10], found in their surveys a significantly better evaluation of the live lectures.

Kalwitzki et al. [12] came to a completely different conclusion: while only 12 of 107 study participants preferred the live lecture, 57 favoured the videos (38 both rated equally well). The different results may be explained by the very different lecture topic in the Tübingen study: This is not about the teaching of exam contents, but about communication patterns in the dental treatment of children and adolescents.

In this study, the question of preference for live or video course comes to the following result: 48% decide for the live, 27% for the video course and 25% are neutral on this question. This result is consistent with most previous studies. In this study, however, the course evaluation gives a different picture than it emerges from the preference: Here, the characteristics of the learning atmosphere, the ability to concentrate, the presence of other students and the acoustical intelligibility in video conferencing are assessed significantly better than live, but vice versa no feature of the live course is judged better than in the video course.

A weakness of the present study is that because of voluntary participation, a selection of the sample cannot be ruled out. But since almost the whole of the year took part in the course, the students of the Göttingen University can be regarded as an almost complete and sufficiently representative sample.

In addition, the course was intended exclusively for candidates in the Second Section of the Medical Examination. The course covers all major clinical subjects, but it conveys theoretical and less clinical practical knowledge. According to Davis et al. [11], results of undergraduate students cannot be transferred to postgraduates, so vice versa the result of the investigation cannot be applied to the pre-clinical study section.

As most of the other investigations on video and live lectures, this study comes to the conclusion that both formats are equally effective.

Video lectures could increase the quality of the teaching, focus the university lectures more on the mediation of research and even improve the practical training through the possibilities of the distance learning. And finally, a video lecture is more expensive in the production than a single live lecture, but it can be repeated as often and for as many students as desired, which would ultimately result in a considerable cost savings.

## References

[1] Nieder GL, Borges NJ, Pearson JC. Medical student use of online lectures: exam performance, learning styles, achievement motivation and gender. Med Sci Educator. 2011;21(3):222–6.

[2] Spickard AI, Alrajeh N, Cordray D, et al Learning about screening using an online or live lecture. J Gen Int Med. 2002;17. 10.1046/j.1525-1497.2002.10731.x PMC1495076 12133144

[3] Cardall S, Krupat E, Ulrich M. Live lecture versus video-recorded lecture: are students voting with their feet? Acad Med. 2008;83(12):1174–1178. 19202495 10.1097/ACM.0b013e31818c6902

[4] Bridge PD, Jackson M, Robinson L. The effectiveness of streaming video on medical student learning: a case study. Med Educ Online. 2009;14:11. 20165525 10.3885/meo.2009.Res00311 PMC2779626

[5] McNulty JA, Hoyt A, Chandrasekhar AJ, et al A three-year study of lecture multimedia utilization in the medical curriculum: associations with performances in the basic sciences. Med Sci Educator. 2011;21(1):29–36.

[6] Paegle RD, Wilkinson EJ, Donnelly MB Videotaped vs traditional lectures for medical studens. Med Educ. 1980;14. 10.1111/j.1365-2923.1980.tb02389.x 7442577

[7] Schreiber BE, Fukuta J, Gordon F Live lecture versus video podcast in undergraduate medical education: a randomised controlled trial. BMC Med Educ. 2010;10:68. 20932302 10.1186/1472-6920-10-68 PMC2958969

[8] Ramlogan S, Raman V, Sweet J A comparison of two forms of teaching instruction: video vs. live lecture for education in clinical periodontology. Eur J Dent Educ. 2014;18(1):31–38. 24423173 10.1111/eje.12053

[9] Fernandes L, Maley MA, Cruickshank C The impact of online lecture recordings on learning outcomes in pharmacology [Refereed article in scholarly journal (C1)]. J Int Assoc Med Sci Educators. 2008;18(2).

[10] Solomon DJ, Ferenchick GS, Laird-Fick HS, et al A randomized trial comparing digital and live lecture formats [ISRCTN40455708 [journal article]. BMC Med Educ. 2004;4(1):1–6. 15569389 10.1186/1472-6920-4-27 PMC535936

[11] Davis J, Crabb S, Rogers E, et al Computer-based teaching is as good as face to face lecture-based teaching of evidence based medicine: a randomised controlled trial. Med Teach. 2008;30. 10.1080/01421590701784349 18484458

[12] Kalwitzki M, Meller C, Beyer C Does teaching method affect students’ perceptions regarding communication patterns in pediatric dentistry? A comparison of lecture and video methods. J Dent Educ. 2011;75(8):1084–1091. 21828302

